# An anoikis-based signature for predicting prognosis in hepatocellular carcinoma with machine learning

**DOI:** 10.3389/fphar.2022.1096472

**Published:** 2023-01-04

**Authors:** Zhang Guizhen, Zhu Weiwei, Wang Yun, Cui Guangying, Zhang Yize, Yu Zujiang

**Affiliations:** ^1^ Department of Infectious Diseases, The First Affiliated Hospital of Zhengzhou University, Zhengzhou, China; ^2^ Gene Hospital of Henan Province, Zhengzhou, China; ^3^ Academy of Medical Sciences, Zhengzhou University, Zhengzhou, China

**Keywords:** hepatocellular carcinoma, anoikis, prognosis, signature, tumor immune microenviroment

## Abstract

**Background:** Hepatocellular carcinoma (HCC) is a common malignancy with high mortality worldwide. Despite advancements in diagnosis and treatment in recent years, there is still an urgent unmet need to explore the underlying mechanisms and novel prognostic markers. Anoikis has received considerable attention because of its involvement in the progression of human malignancies. However, the potential mechanism of anoikis-related genes (ANRGs) involvement in HCC progression remains unclear.

**Methods:** We use comprehensive bioinformatics analyses to determine the expression profile of ANRGs and their prognostic implications in HCC. Next, a risk score model was established by least absolute shrinkage and selection operator (Lasso) Cox regression analysis. Then, the prognostic value of the risk score in HCC and its correlation with clinical characteristics of HCC patients were further explored. Additionally, machine learning was utilized to identify the outstanding ANRGs to the risk score. Finally, the protein expression of DAP3 was examined on a tissue microarray (TMA), and the potential mechanisms of DAP3 in HCC was explored.

**Results:** ANRGs were dysregulated in HCC, with a low frequency of somatic mutations and associated with prognosis of HCC patients. Then, nine ANRGs were selected to construct a risk score signature based on the LASSO model. The signature presented a strong ability of risk stratification and prediction for overall survival in HCC patients.Additionally, high risk scores were closely correlated with unfavorable clinical features such as advanced pathological stage, poor histological differentiation and vascular invasion. Moreover, The XGBoost algorithm verified that DAP3 was an important risk score contributor. Further immunohistochemistry determined the elevated expression of DAP3 in HCC tissues compared with nontumor tissues. Finally, functional analyses showed that DAP3 may promote HCC progression through multiple cancer-related pathways and suppress immune infiltration.

**Conclusion:** In conclusion, the anoikis-based signature can be utilized as a novel prognostic biomarker for HCC, and DAP3 may play an important role in the development and progression of HCC.

## Introduction

Hepatocellular carcinoma (HCC) is the most common subtype of primary liver cancer, ranking sixth in cancer incidence and third in cancer-related mortality worldwide (Sung et al., 2021). Although major advances have been made recently in diagnosis and treatment, such as surgery, chemotherapy and immunotherapy, the prognosis of HCC is far from satisfactory (Kulik and El-Serag, 2019). Therefore, identifying novel prognostic markers to improve the outcomes of HCC patients is still much necessary, and may contribute to optimizing personalized treatment of HCC.

Anoikis is a type of programmed cell death that occurs upon cell detachment from the native extracellular matrix (ECM) (Taddei et al., 2012). Under physiological conditions, anoikis can efficiently remove displaced cells and prevent detached cells from attaching to other tissues. Thus, anoikis plays an important role in tissue homeostasis and development (Taddei et al., 2012; Tajbakhsh et al., 2019). Unfortunately, anoikis is also implicated in pathological processes (Michel, 2003). It was reported that the anoikis of vascular cells was enhanced during cardiovascular infections, thereby contributing to pathological remodeling of cardiovascular tissues (Beaufort et al., 2011). Yin et al. (2022) demonstrated that anoikis resistance promotes fibroblast activation and is involved in pulmonary fibrosis. Moreover, anoikis is closely correlated with the development of human cancers. Survival in the in circulatory system and/or distant organs in the absence of native ECM contacts is a pivotal step for cancer metastasis. Therefore, anoikis resistance is the theoretical prerequisite for the aggressive metastasis of malignancies. The dysregulation of ANRGs has been found in various cancers such as gastric, breast and lung cancer (Madajewski et al., 2016; Du et al., 2018; Du et al., 2021). NOX4 overexpression could promote anoikis resistance in gastric cancer by increasing reactive oxygen species (ROS) generation and upregulating EGFR expression, thereby enhancing the metastasis of gastric cancer (Du et al., 2018). Additionally, anoikis resistance was shown to exert an important function in therapeutic resistance (Sharma et al., 2022). Although anoikis plays a non-negligible role in HCC progression (Hu et al., 2017; Li et al., 2019; Mo et al., 2020; Song et al., 2021), its prognostic value has not been systematically evaluated in HCC.

In this study, we investigated the alterations of ANRGs, explored their prognostic values in HCC and further developed a nine-gene signature that could predict the prognosis of HCC patients. Moreover, we determined the expression of DAP3 protein in an HCC tissue microarray. DAP3 was speculated to be an oncogene involved in the progression of HCC.

## Materials and methods

### Datasets

The TCGA-HCC cohort, including the mutations, RNA expression profiles and clinical data of 50 non-tumor samples and 370 HCC samples, was utilized for identification. A total of 203 HCC samples from HCCDB were selected as the validation cohort.

### Tissue samples

A tissue microarray (TMA) containing 80 paired adjacent non-tumor and HCC tissues was constructed by our laboratory. Our study was approved by the Ethics Committee of the First Affiliated Hospital of Zhengzhou University.

### Immunohistochemistry

IHC and evaluation were conducted as previously reported (Cui et al., 2019; Li et al., 2020). Two experienced pathologists blinded to the clinicopathological data separately scored the immunostaining samples. The criteria of the score according to the proportion of positive cells were as follows: none, 0; <25%, 1; 25%–50%, 2; 50%–75%, 3; 75%–100%, 4. The intensity of staining was evaluated as follows: no staining, 0; weak, 1; moderate, 2; and strong, 3. The total score was calculated by multiplying these two subscores. Specimens with scores of 0–4 were classified as low expression, whereas scores of 5–8 and 9–12 were defined as moderate and high expression, respectively. Antibody information is listed in Supplementary Table S1.

### Identification and validation of the risk score model

Univariate Cox regression analysis was performed to select overall survival (OS)-related ANRGs, followed by LASSO regression analysis to develop the prognostic model. To prevent the overfitting effect of the model, we determined the parameter λ by tenfold cross validations. Based on the optimal λ value and the corresponding coefficients, nine ANRGs were screened to construct the risk score model. The risk score for each patient was calculated using the following formula:

$$Risk\;Score=\sum_{i=1}^{n}Coefi*Expi$$

Coefi and Expi represent the coefficient and expression level of each selected gene, respectively. Based on the median score, patients were divided into low- and high-risk groups. Finally, Kaplan-Meier survival curves were used to evaluate the prognostic capacity of this risk model. Additionally, validation was conducted in the validation cohort.

### Pathway enrichment analysis

We performed Kyoto Encyclopedia of Genes and Genomes (KEGG) analysis, Gene Ontology (GO) and Gene Set Enrichment Analysis (GSEA) using R packages to identify the potential mechanisms and related pathways of anoikis regulators in HCC.

### Machine learning algorithm

The machine learning algorithm, extreme gradient boosting (XGBoost) with the SHapley Aditive exPlanation (SHAP) method was utilized to investigate the importance of ANRGs in the risk score model.

### Statistical analysis

R language was utilized for the statistical analyses. To screen differentially expressed ANRGs in HCC, we performed differentiation analysis by limma in R software (log_2_|fold change| >1.0, *p* < 0.05 and adjust *p* < 0.05). The differences between two groups were determined by Student’s t-test or Chi-square test. Univariate Cox regression analyses were performed to identify prognostic regulators. Kaplan-Meier and log-rank tests were implemented to analyze the survival time of patients. *p* < 0.05 was considered statistically significant.

## Results

### Differential expression and prognostic value of anoikis-related genes in hepatocellular carcinoma

**FIGURE 1:**
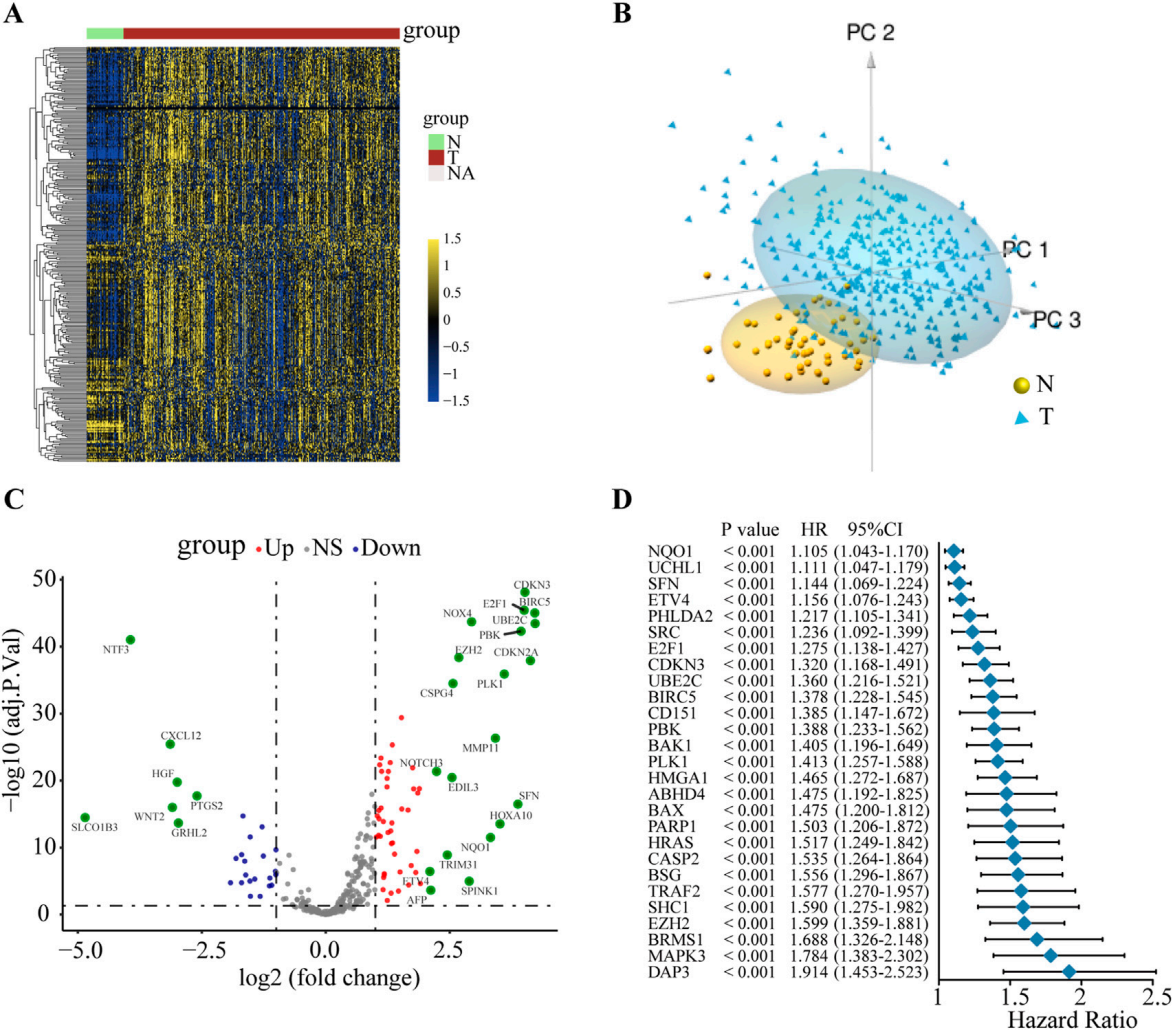
The variation and prognostic value of anoikis-related genes in HCC. **(A)** Heat map of ANRGs of non-tumor tissues and HCC tissues. **(B)** Three-dimensional PCA of the non-tumor samples and tumor samples. **(C)** The volcano diagram of 311 differentially expressed ANRGs. Red dots: upregulation. Purple dots: downregulation. Gray dots: no significant difference. **(D)** Univariate Cox analysis of OS in HCC. ANRGs: anoikis-related genes; PCA: principal component analysis; HR: hazard ratio. CI: confidence interval; OS: overall survival.

To explore the potential mechanism of ANRGs in HCC, we first investigated the mRNA expression profiles of 311 ANRGs in HCC based on the TCGA database. The heatmap showed that these regulators were generally dysregulated in the tumor tissues compared with the non-tumor tissues (Figure 1A). Principal component analysis (PCA) indicated that gene expression profiles between tumor and non-tumor tissues were well differentiated (Figure 1B, Additional file 1: Supplementary Figure S1). To further gain insights into the relevant genetic alterations, we investigated the somatic mutations of ANRGs in HCC. The results demonstrated that only 26 of the 368 patients (7.07%) had somatic mutations (Additional file 1: Supplementary Figure S2), suggesting that somatic mutations may not be the only factor responsible for the dysregulation of anoikis regulators, which needs to be further explored. Subsequently, a total of 178 differentially expressed genes (DEGs) between HCC tissues and non-tumor tissues were identified (Figure 1C). Among them, most regulators were upregulated. The forest plot shows the top 27 ANRGs that were statistically correlated with OS (*p* < 0.001) (Figure 1D). Collectively, these results suggested that anoikis regulators were generally dysregulated in HCC and had potential prognostic value in HCC.

### Identification and validation of the risk score signature of anoikis regulators

**FIGURE 2:**
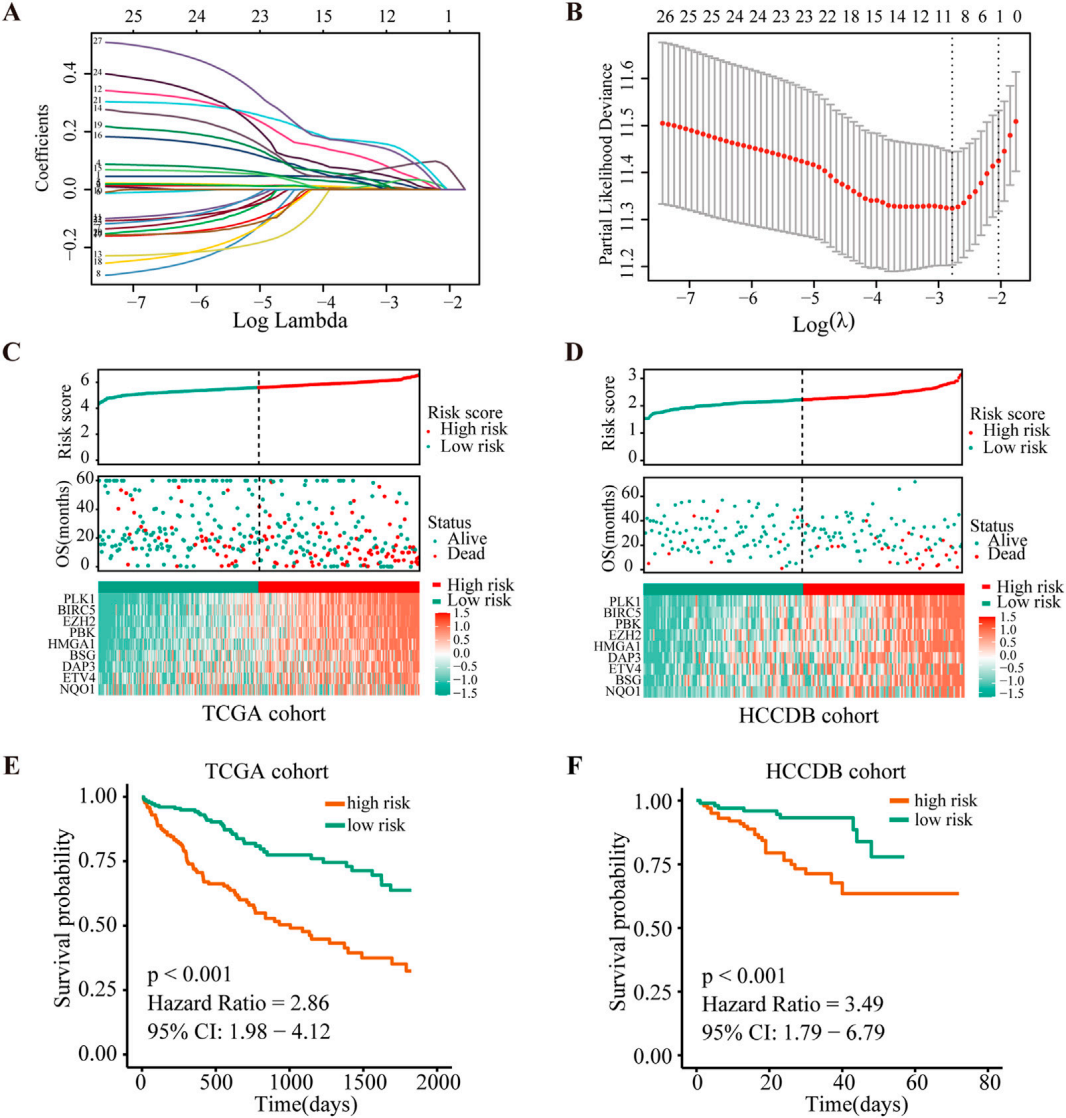
Construction and validation of the risk score signature of anoikis-related genes. **(A)** Selection of the optimal parameter (lambda) in the LASSO model. **(B)**. LASSO coefficients of the 27 ANRGs in TCGA cohort. **(C,D)** Overall survival analysis for high-risk and low-risk groups in the training (TCGA) cohort and validation (HCCDB) cohort, respectively. **(E,F)** Diagrams of the risk score, survival status and heatmap for nine model genes in the TCGA and HCCDB cohorts.

As a next step, we performed LASSO Cox regression analysis to further compress these 27 significant ANRGs, thereby reducing the number of genes in the model. Then, a risk score signature was established by tenfold cross-validation (Figure 2A) and the value of λ was determined according to the minimum partial likelihood deviation (Figure 2B). Nine genes (NQO1, ETV4, BSG, HMGA1, DAP3, PBK, BIRC5, PLK1, and EZH2) were ultimately selected for the signature (Figure 2B). The Kaplan-Meier analysis revealed that higher expression of these nine regulators indicated unfavorable prognosis in the TCGA cohort (Additional file 1: Supplementary Figure S3).The risk score of each sample in the TCGA cohort was calculated with the following formula: Risk Score = (0.01964572 × NQO1 expression) + (0.001610727 × ETV4 expression) + (0.141016275 × BSG expression) + (0.031793916 × HMGA1 expression) + (0.120024309 × DAP3 expression) + (0.058951788 × PBK expression) + (0.005813196 × BIRC5 expression) + (0.081335647 × PLK1 expression) + (0.036663928 × EZH2 expression).

To further determine the prognostic value of the signature, we stratified patients from the TCGA-HCC cohort into low- or high-risk groups according to the median score. Figure 2C depicts the distribution of risk scores of patients with HCC and the relationship between risk scores and survival states. Survival analysis demonstrated that patients with high risk scores tended to have shorter OS times than patients with low risk scores (Figure 2E). To determine the robustness of this nine-gene signature, we conducted the same analysis in the HCCDB validation cohort (n = 203). Encouragingly, the results were consistent with the aforementioned findings in the TCGA-HCC cohort (Figures 2D,F, Additional file 1: Supplementary Figure S4). These findings suggested that the anoikis-based signature had good performance and could be utilized as an effective prognostic tool for HCC patients.

### Correlations between the risk score and clinical characteristics in hepatocellular carcinoma patients

**FIGURE 3:**
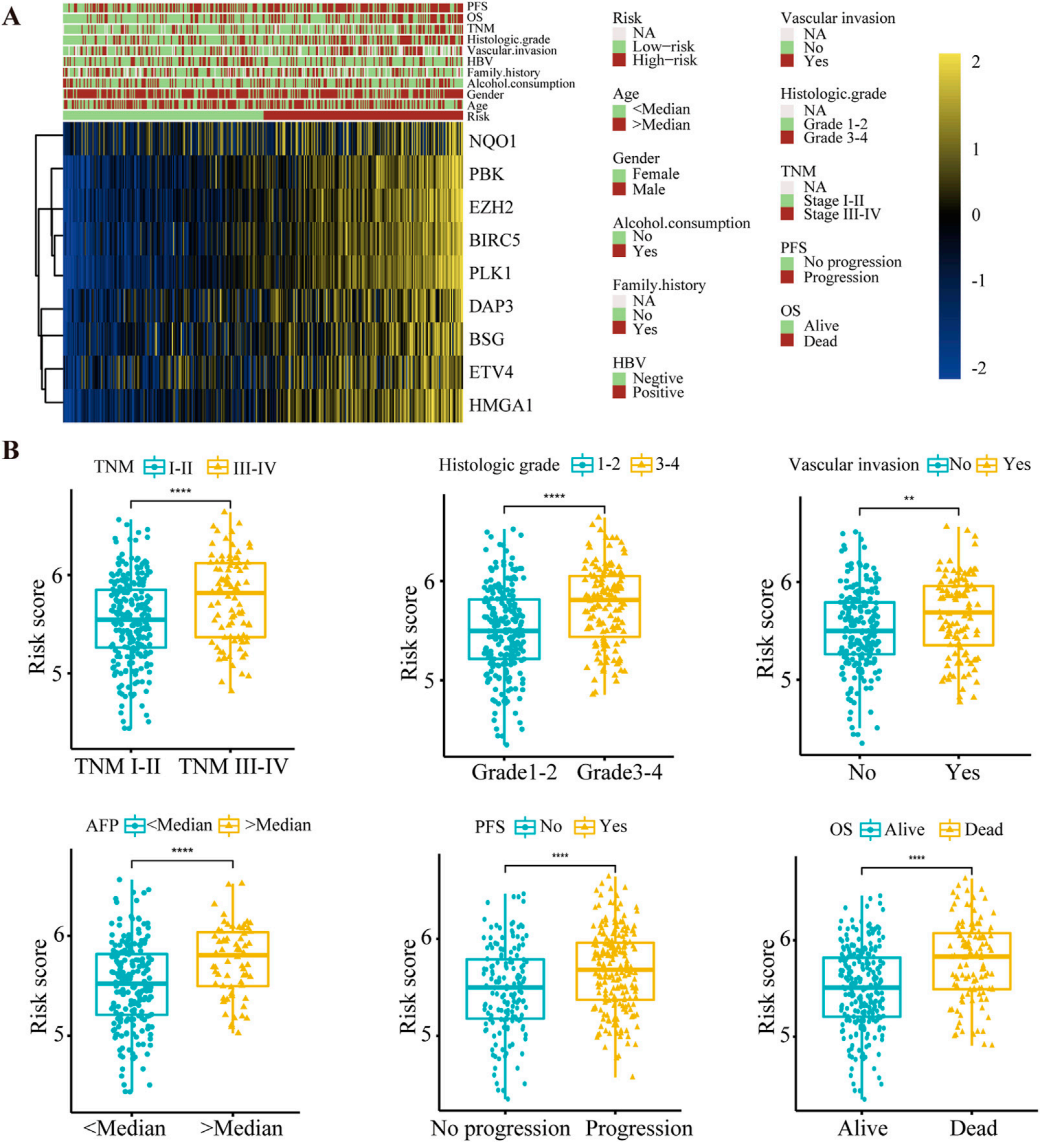
The relationship between the risk score and clinical characteristics in HCC patients. **(A)** Heat map of nine anoikis-related genes expression and corresponding clinicopathological features of low- and high-risk group. **(B)** The relationships between the risk score and clinical characteristics including TNM stage, histologic grade, vascular invasion, AFP level, PFS and OS. **p < 0.01, ****p < 0.0001. TNM, Tumor Node Metastasis; PFS, Progression-Free Survival; OS, Overall Survival.

Subsequently, correlations between the risk score and clinical characteristics were investigated. The expression of nine anoikis regulators and clinical features of HCC patients in the TCGA database were visualized by a heatmap according to risk status (Figure 3A). Further analysis illustrated that advanced TNM stage, poor histological differentiation, vascular invasion and high levels of AFP indicated a higher risk score. Additionally, differential risk scores were observed in patients with progression or a poor OS (Figure 3B, Supplementary Table S2). Our findings suggested that the risk score had significant relationships with the malignancy of HCC.

### Contributions of ANRGs to the risk score by machine learning

**FIGURE 4:**
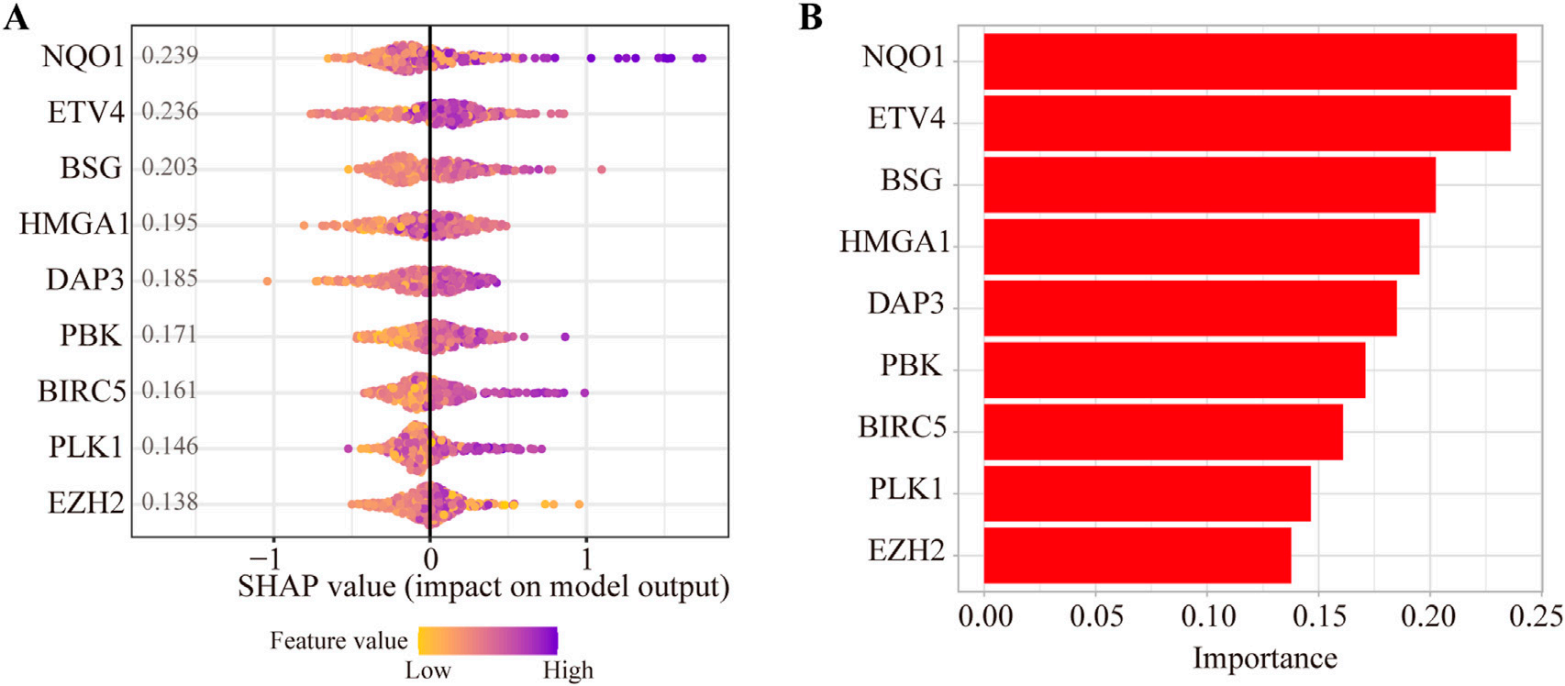
Contributions of anoikis-related genes to the risk score by machine learning. **(A)** The rank of nine anoikis-related genes according to the SHAP value. **(B)** Important feature score in the XGBoost algorithm model.

To evaluate the contributions of nine ANRGs to the risk score, we established a model as the classifier by applying the XGBoost algorithm. Figure 4A shows the rank of nine ANRGs based on the SHAP value. Each dot represents a value of the specific gene. By summing all scores of the samples, the regulators were sorted according to the final scores (Figure 4B).

### DAP3 was upregulated in hepatocellular carcinoma tissues

**FIGURE 5:**
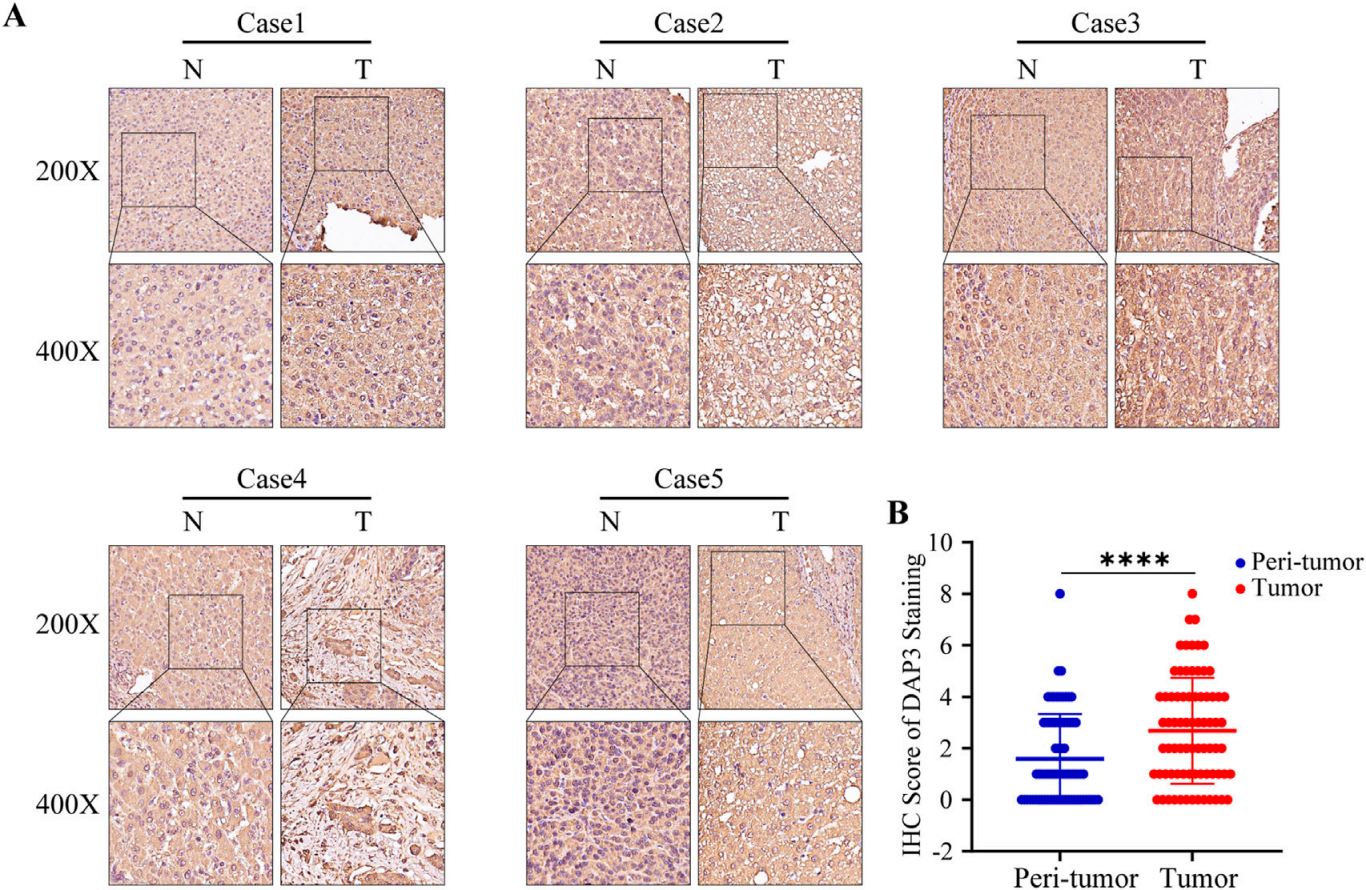
DAP3 was upregulated in HCC tissues. **(A)** Representative images of DAP3 staining in adjacent normal tissues and HCC tissues. **(B)** The comparison of IHC score between adjacent normal tissues and HCC tissues.

Previous studies have proven that NQO1, ETV4, BSG, and HMGA1 are highly expressed in HCC (Jin et al., 2019; Teng et al., 2019; Yang et al., 2021; Zheng et al., 2022). In addition, the upregulation of PBK, BIRC5, PLK1, and EZH2 was also validated in HCC tissues (Xiao et al., 2019; Yang et al., 2019; Tian et al., 2020; Huang et al., 2021; Lin et al., 2021; Peng et al., 2021). However, the protein expression of DAP3 in HCC has scarcely been investigated, so we selected DAP3 as the candidate molecule. Subsequently, an IHC assay was conducted to explore the expression of DAP3 in HCC tissues. DAP3 protein was mainly localized to the nucleus. As shown in Figures 5A,B, DAP3 was significantly upregulated in HCC tissues compared with adjacent non-tumor tissues which was consistent with the mRNA expression in TCGA.

### Functional analysis of DAP3

**FIGURE 6:**
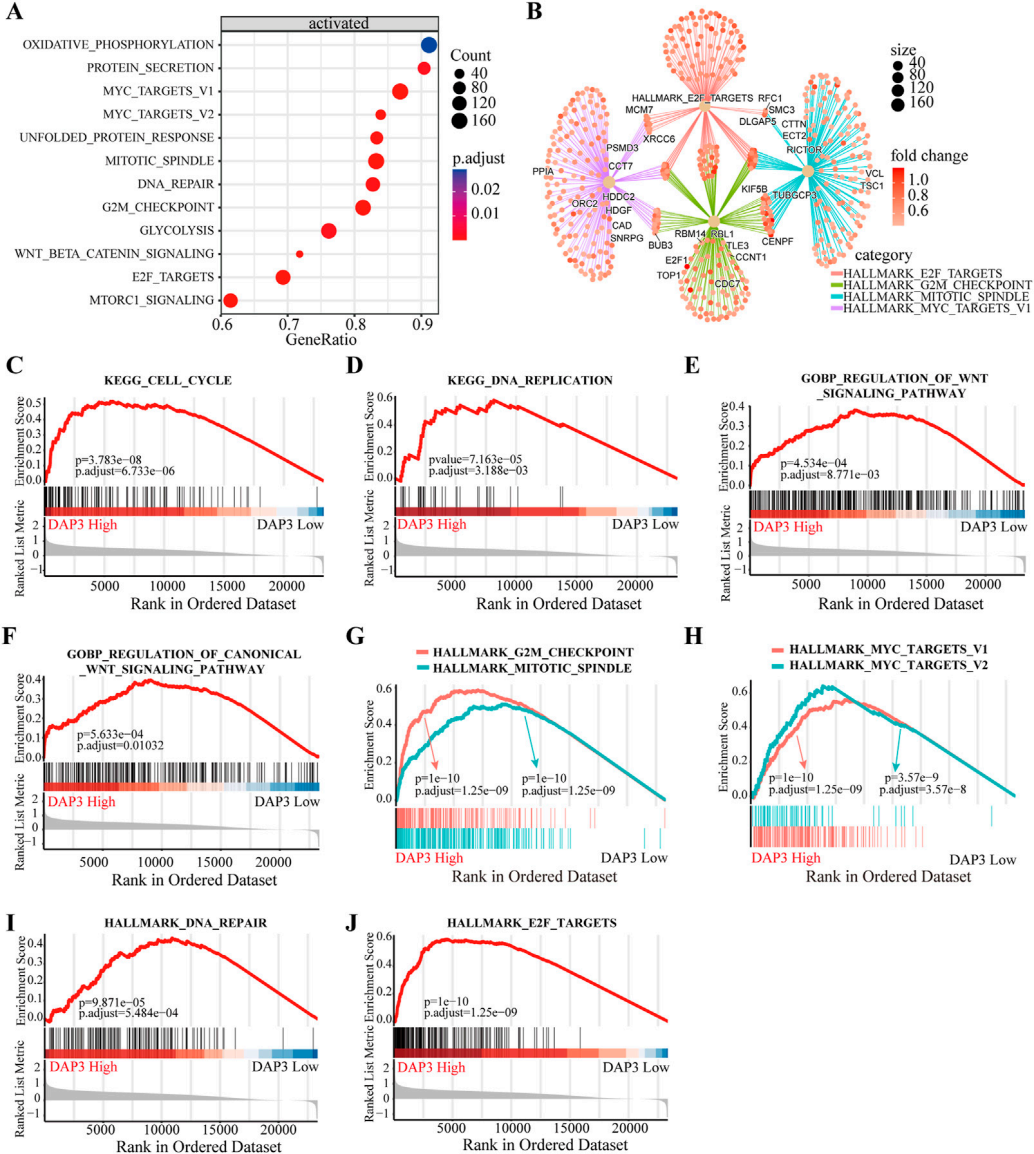
Functional annotation of different subgroups. **(A)** KEGG enrichment pathways analysis of differentially expressed genes in DAP3 high expression group. **(B)** Interactions of multiple pathways. **(C,D)** KEGG enrichment and GO analysis **(E,F)** showed that the “cell cycle,” “DNA replication” and “Wnt signaling” pathways were significantly enriched in DAP3 high expression group. **(G–J)** The GSEA analysis between DAP3 low and high expression groups using hallmark gene sets.

To deeply investigate the potential roles of DAP3 in HCC, we performed KEGG pathway analysis based on DEGs between subgroups with differential DAP3 expression**.** The results indicated that multiple pathways involved in cell proliferation and metabolism were significantly activated in the group with high DAP3 expression (Figure 6A). Figure 6B illustrates the interactions of molecules in these pathways, implying that DAP3 may exert an oncogenic role in HCC mainly through the “HALLMARK_E2F_TARGETS,” “HALLMARK_G2M_CHECKPOINT,” “HALLMARK_MITOTIC_SPINDLE” and “HALLMARK_MYC_TARGETS” pathways. As shown in Figures 6C–F, “cell cycle,” “DNA replication” and “Wnt signaling pathways” were significantly enriched when DAP3 was upregulated. GSEA demonstrated that “G2M checkpoint,” “Mitotic spindle,” Myc targets,” “DNA repair” and “E2F targets,” which are related to cancer development and progression, were significantly activated in patients with high DAP3 expression (Figures 6G–J). These results indicated that DAP3 may serve as an oncogene in HCC.

### The correlation between immune infiltration and DAP3

**FIGURE 7:**
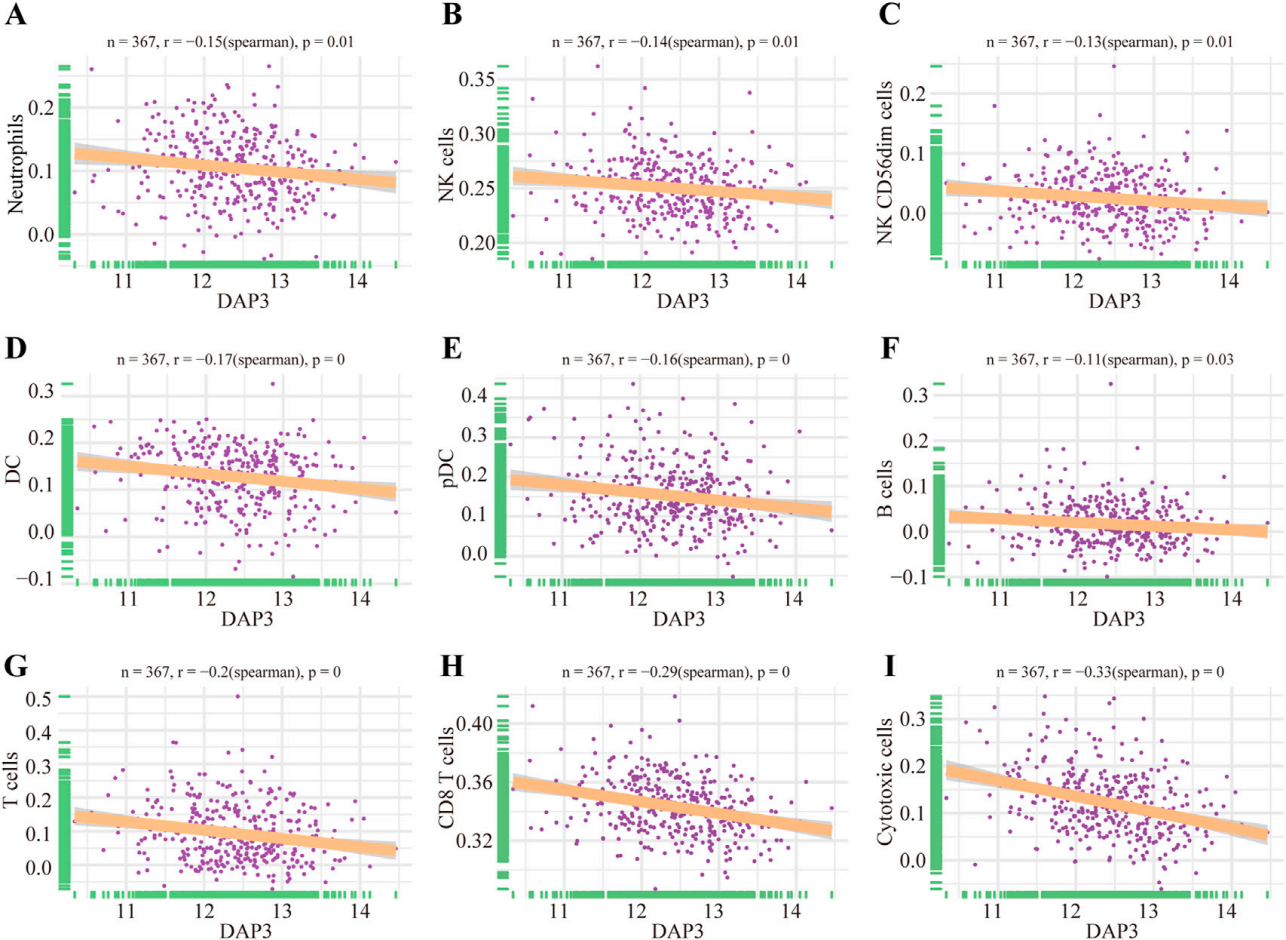
Immune infiltration analysis of DAP3. **(A–E)** Correlation between innate immune cells and the expression of DAP3, including DC, NK cells, and cells. **(F–I)** The infiltration of adaptive immune cells including B cells, T cells, CD8^+^T cells and cytotoxic cells, was negatively correlated with the expression of DAP3. DC: dendritic cells; NK cells: natural killer cells.

As mentioned above, Wnt signaling pathways were apparently enriched in the group with high DAP3 expression. Moreover, recent studies have shown that Wnt pathways are implicated in regulating immune infiltration of the tumor microenvironment. (Chae and Bothwell, 2018; Li et al., 2021; Takeuchi et al., 2021; Du et al., 2023). Therefore, we further explored whether DAP3 expression was related to immune infiltration in HCC based on the TCGA database. The results showed that innate immune cells, including neutrophils, dendritic cells, and natural killer cells, were negatively correlated with DAP3 expression (Figures 7A–E). Moreover, the infiltration of adaptive immune cells such as B cells, T cells, CD8^+^ T cells and cytotoxic cells, which are responsible for the antitumor response, was significantly inhibited in patients with high DAP3 expression (Figures 7F–I). These findings implied that DAP3 expression might be associated with the immunosuppressive tumor microenvironment of HCC.

## Discussion

HCC is a highly life-threatening malignancy, and effective predictive biomarkers of HCC are still lacking. Recently, an increasing number of studies have demonstrated alterations of anoikis regulators and their significant role in tumorigenesis and progression in various malignancies, including lung cancer, prostate cancer, breast cancer and glioblastoma (Jin et al., 2018; Tajbakhsh et al., 2019; Du et al., 2021; Sun et al., 2022; Yu et al., 2022). However, the underlying mechanisms and prognostic value of dysregulated anoikis-related genes in HCC remain unclear. Hence, we explored the alterations of ANRGs and constructed a risk score signature to predict the prognosis in HCC.

In this study, we first compared the expression profiles of 311 ANRGs retrieved from the TCGA database in HCC tissues and non-tumor tissues. The results showed that ANRGs were significantly differentially expressed, and PCA based on these dysregulated genes could distinguish tumor and non-tumor samples well. Additionally, we investigated whether somatic mutations contributed to aberrant expression of ANRGs, but few patients (7.07%) exhibited mutations, implying that there may be other regulatory mechanisms that led to alterations in ANRGs. Given the prognostic value of ANRGs evaluated by univariate Cox analysis, we established a nine-gene risk score signature, with NQO1, ETV4, BSG, HMGA1, DAP3, PBK, BIRC5, PLK1, and EZH2, by means of the scoring algorithm to predict HCC patient prognosis. Further analysis suggested that the risk score model had stable capabilities of stratification and prediction for OS in HCC patients. In addition, we found that high risk scores were closely associated with advanced TNM stage, poor histological differentiation, vascular invasion and high levels of AFP. These findings revealed that the anoikis-based gene signature may serve as a potential tool to predict the progression and prognosis in HCC patients.

To obtain more insights into the risk score model, we conducted machine learning *via* the XGBoost algorithm. According to the contributions of ANRGs, genes were ranked with NQO1, ETV4, BSG, HMGA1, and DAP3 as the top 5 factors. We further identified the elevated expression of DAP3 in HCC and found that DAP3 may be involved in HCC progression by participating in multiple cancer-related pathways, including the cell cycle, DNA replication and Wnt signaling pathways. Notably, emerging data have demonstrated that the activation of Wnt/β-catenin signaling contributes to tumor progression by modulating the infiltration of immune cells into the tumor microenvironment (Swafford and Manicassamy, 2015; Takeuchi et al., 2021). For instance, Marina et al. found that Wnt/β-catenin could promote immune escape in HCC by decreasing the recruitment of dendritic cells and consequently impairing T-cell activity (Ruiz de Galarreta et al., 2019). Although Wnt pathways were activated in response to elevated DAP3, it was not clear whether upregulation of DAP3 was associated with immune infiltration. Interestingly, our study found that there was a negative correlation between immune infiltration and DAP3 expression in HCC.

Previous studies have revealed that NQO1 is upregulated and promotes an aggressive phenotype in HCC (Shimokawa et al., 2020; Yang et al., 2021; Wang et al., 2022). Zheng et al. reported that the enhanced expression of ETV4 by HBx may stimulate the metastasis of HCC (Zheng et al., 2022) The literature showed that the interaction between ETV4 and YAP conferred a growth advantage to HCC and had a protumorigenic role (Xu et al., 2022). Some studies have shown that BSG and HMGA1 play significant roles in the development and progression of HCC (Li et al., 2015; Jin et al., 2019; Teng et al., 2019; Chen et al., 2022). Moreover, elevated expression of PBK, BIRC5, PLK1 and EZH2 was identified in HCC tissues (Xiao et al., 2019; Yang et al., 2019; Tian et al., 2020; Lin et al., 2021; Peng et al., 2021). It was reported that DAP3 was upregulated in pancreatic cancer, breast cancer and correlated with malignant phenotypes (Wazir et al., 2015; Sui et al., 2021). Han et al. elaborated that DAP3 could suppress A-to-I RNA editing in cancer cells and promote cancer progression (Han et al., 2020). However, studies on the expression and potential function of DAP3 in HCC are very limited, and these issues remain to be investigated. Our work as a pioneering study may provide insights into the role of DAP3 in HCC progression.

However, there are some limitations and shortcomings in our study. First, we identified nine anoikis-related genes (NQO1, ETV4, BSG, HMGA1, DAP3, PBK, BIRC5, PLK1, and EZH2) in the risk score model, but only DAP3 protein expression was validated by the tumor microarray. More attention needs to be paid to other molecules in further research. Second, we only investigated the potential function of DAP3 based on bioinformatic analysis. Research on the role of DAP3 in promoting HCC progression is worth conducting in the future.

In conclusion, we developed and validated a risk score signature to predict the prognosis of HCC patients based on dysregulated anoikis-related genes. DAP3 is an important tumor-promoting molecule in HCC. This study provides a unique method for the application of new prognostic biomarkers for HCC.

## Data Availability

The raw data supporting the conclusion of this article will be made available by the authors, without undue reservation.
