# Supplementary material for: An anoikis-based signature for predicting prognosis in hepatocellular carcinoma with machine learning
Source: Front Pharmacol. 2023 Jan 4;13:1096472. doi: 10.3389/fphar.2022.1096472 (PMC9846167; doi:10.3389/fphar.2022.1096472)
Supplement: Supplementary file 2 [file DataSheet1.pdf]

Supplementary Material

1 Supplementary Figures

1.1 Supplementary Figure 1

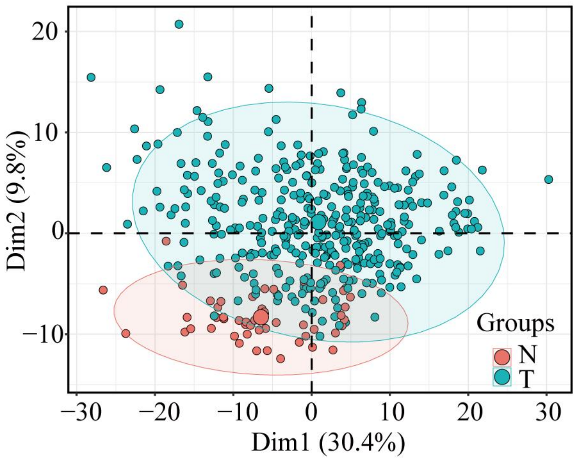

Supplementary Figure 1. Two-dimensional PCA of the non-tumor samples and tumor samples. PCA: principal component analysis.

1.2 Supplementary Figure 2

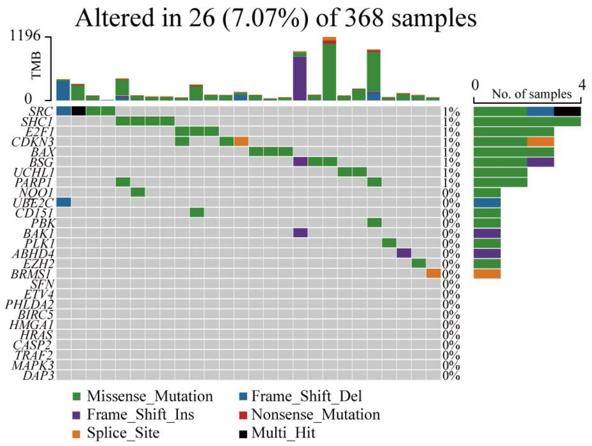

Supplementary Figure 2. The somatic mutation frequency in anoikis-related genes in the TCGA-HCC cohort.

1.3 Supplementary Figure 3

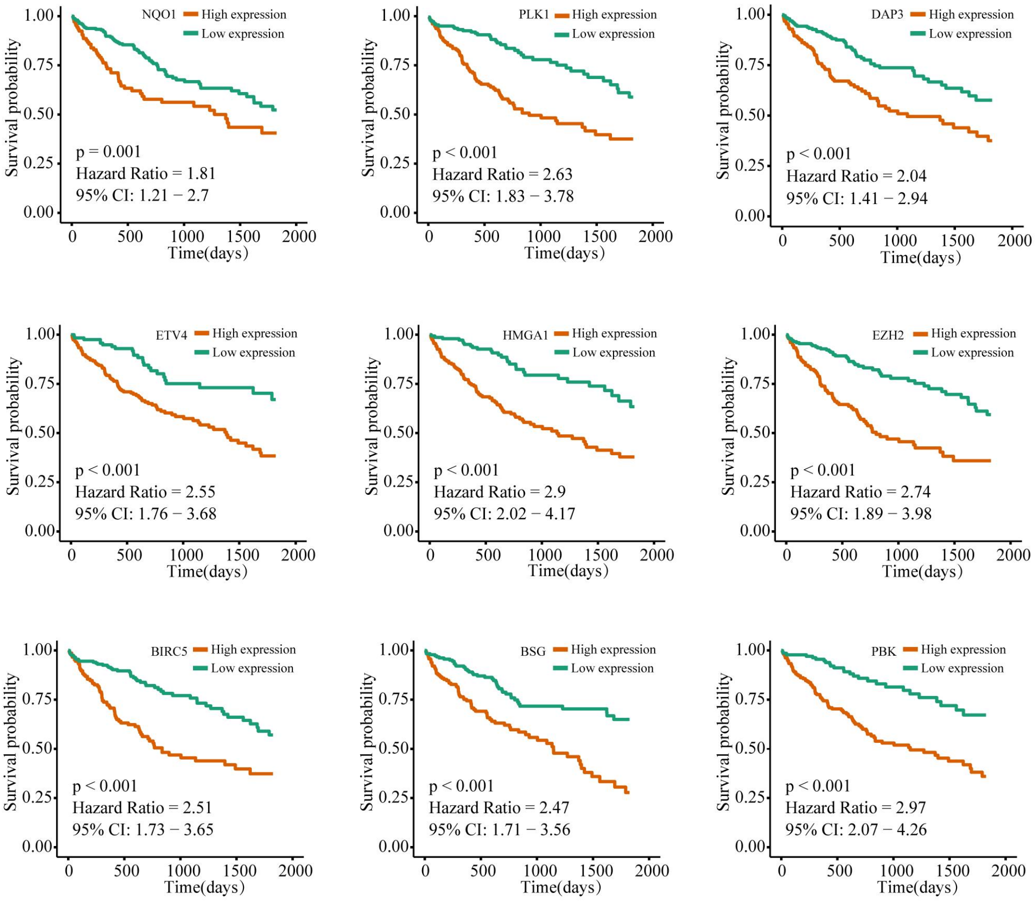

Supplementary Figure 3. Survival analysis of nine anoikis-related genes for patients with HCC in the TCGA training cohort.

1.4 Supplementary Figure 4

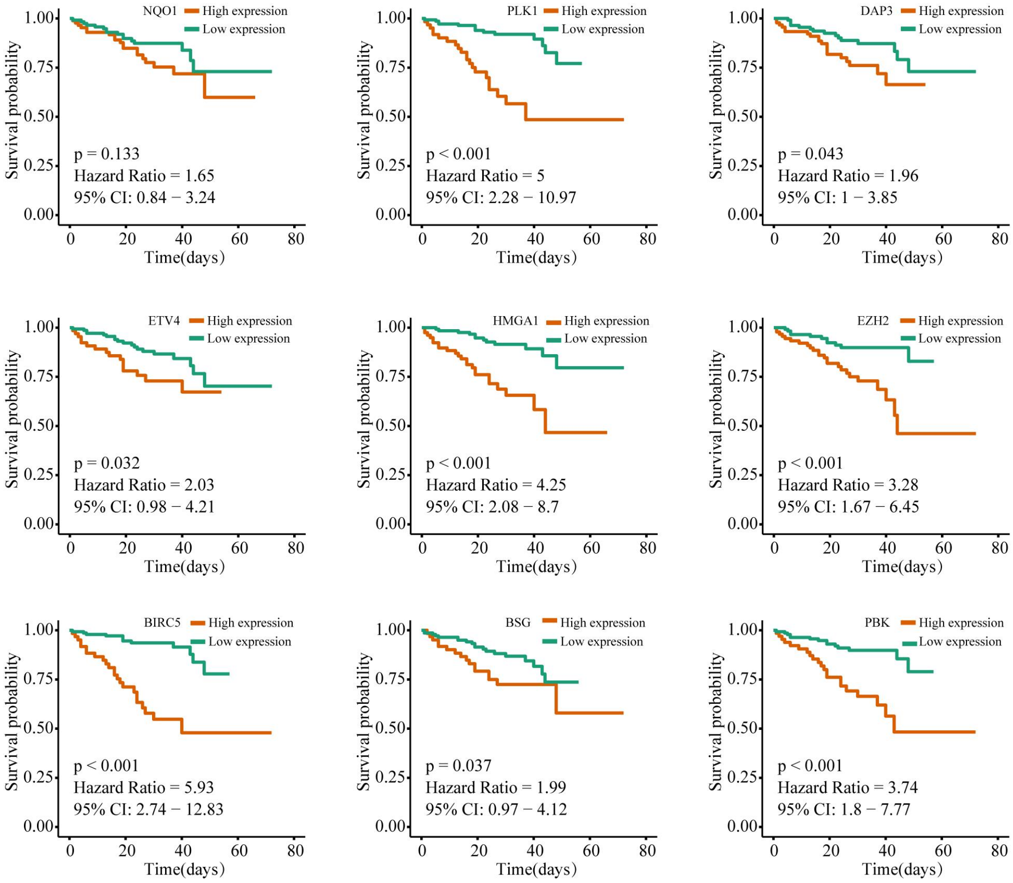

Supplementary Figure 4. Overall survival analysis of nine anoikis-related genes for patients with HCC in the HCCDB validation cohort.
